# Aspartyl peptidase May1 induces host inflammatory response by altering cell wall composition in the fungal pathogen *Cryptococcus neoformans*

**DOI:** 10.1128/mbio.00920-24

**Published:** 2024-05-14

**Authors:** Yeqi Li, Benjamin Chadwick, Tuyetnhu Pham, Xiaofeng Xie, Xiaorong Lin

**Affiliations:** 1 Department of Microbiology, University of Georgia, Athens, Georgia, USA; 2 Department of Plant Biology, University of Georgia, Athens, Georgia, USA; The University of British Columbia, Vancouver, British Columbia, Canada

**Keywords:** carbon dioxide, pH sensing, chitosan, chitin synthase, chitin deacetylase, inflammation, cryptococcosis

## Abstract

*Cryptococcus neoformans* causes cryptococcal meningoencephalitis, a disease that kills more than 180,000 people annually. Contributing to its success as a fungal pathogen is its cell wall surrounded by a capsule. When the cryptococcal cell wall is compromised, exposed pathogen-associated molecular pattern molecules (PAMPs) could trigger host recognition and initiate attack against this fungus. Thus, cell wall composition and structure are tightly regulated. The cryptococcal cell wall is unusual in that chitosan, the acetylated form of chitin, is predominant over chitin and is essential for virulence. Recently, it was shown that acidic pH weakens the cell wall and increases exposure of PAMPs partly due to decreased chitosan levels. However, the molecular mechanism responsible for the cell wall remodeling in acidic pH is unknown. In this study, by screening for genes involved in cryptococcal tolerance to high levels of CO_2_, we serendipitously discovered that the aspartyl peptidase May1 contributes to cryptococcal sensitivity to high levels of CO_2_ due to acidification of unbuffered media. Overexpression of *MAY1* increases the cryptococcal cell size and elevates PAMP exposure, causing a hyper-inflammatory response in the host while *MAY1* deletion does the opposite. We discovered that May1 weakens the cell wall and reduces the chitosan level, partly due to its involvement in the degradation of Chs3, the sole chitin synthase that supplies chitin to be converted to chitosan. Consistently, overexpression of *CHS3* largely rescues the phenotype of *MAY1*^oe^ in acidic media. Collectively, we demonstrate that May1 remodels the cryptococcal cell wall in acidic pH by reducing chitosan levels through its influence on Chs3.

**IMPORTANCE:**

The fungal cell wall is a dynamic structure, monitoring and responding to internal and external stimuli. It provides a formidable armor to the fungus. However, in a weakened state, the cell wall also triggers host immune attack when PAMPs, including glucan, chitin, and mannoproteins, are exposed. In this work, we found that the aspartyl peptidase May1 impairs the cell wall of *Cryptococcus neoformans* and increases the exposure of PAMPs in the acidic environment by reducing the chitosan level. Under acidic conditions, May1 is involved in the degradation of the chitin synthase Chs3, which supplies chitin to be deacetylated to chitosan. Consistently, the severe deficiency of chitosan in acidic pH can be rescued by overexpressing *CHS3*. These findings improve our understanding of cell wall remodeling and reveal a potential target to compromise the cell wall integrity in this important fungal pathogen.

## INTRODUCTION

*Cryptococcus neoformans* is an encapsulated basidiomycete fungus often found in soil, trees, and bird guano (1, 2). Exposure to this environmental microbe through inhalation in the general population results in either clearance or dormancy in the lungs (3–5). In immunodeficient hosts, however, the fungus often spreads from the lungs to other organs with a predilection to the brain, causing cryptococcal meningoencephalitis (6). Cryptococcal meningoencephalitis is fatal without treatment. It kills ~180,000 people each year and is responsible for 15%–19% of deaths of people living with HIV/AIDS (7–9). The threat of this fungus to public health has prompted WHO to recently list it as a fungal pathogen of the top critical priority in need of further research on its pathogenesis, diagnosis, and therapy (10, 11).

The fungal cell wall is essential for maintaining cell morphology and for protecting the fungus from various host or environmental insults (12, 13). An indispensable component of the fungal cell wall is chitin, which contributes to the strength and integrity of the cell wall (14, 15). Chitin, a linear polymer of β-(1,4)-linked N-acetylglucosamine (GlcNAc), is synthesized by chitin synthases (14–16). Chitosan is the deacetylated derivative of chitin generated by chitin deacetylases (17, 18). The *C. neoformans* cell wall differs from other yeasts in that chitosan dominates over chitin under laboratory growth conditions and in infected mouse lungs (17, 18). Chitosan levels can be drastically reduced either by deleting the chitin synthase gene *CHS3*, which supplies chitin for deacetylation, or by deleting all three *CDA1-3* genes that encode chitin deacetylases (17, 19). Recently, Upadhya and colleagues observed a 90% reduction of chitosan due to decreased medium pH when *C. neoformans* cells were grown in unbuffered yeast nitrogen base (YNB) medium (20). These cryptococcal cells exhibited altered cell wall architecture and showed reduced virulence in a murine model compared with cells grown in buffered YNB medium (20). How acidic conditions cause chitosan deficiency in this fungus is unknown.

Our previous work found that CO_2_ tolerance is an important virulence trait for *C. neoformans* (21–24). In our search for genes involved in cryptococcal tolerance to high levels of CO_2_, which acidified the unbuffered media, we serendipitously discovered the role of May1, an aspartyl protease, in cell wall remodeling by interfering the function of Chs3 under acidic conditions. We found that *MAY1* overexpression increased PAMP exposure and decreased chitosan levels in the cell wall, which then elicited exacerbated inflammatory response *in vivo*. Overexpression of *CHS3*, but not the chitosan deacetylase genes *CDA1-3*, rescued the chitosan deficiency-associated phenotypes of *MAY1*^oe^ in acidic pH, suggesting that May1 primarily affects the supply of chitin for conversion to chitosan.

## RESULTS

### The loss of May1 enhances cryptococcal CO_2_ tolerance

**Fig 1:**
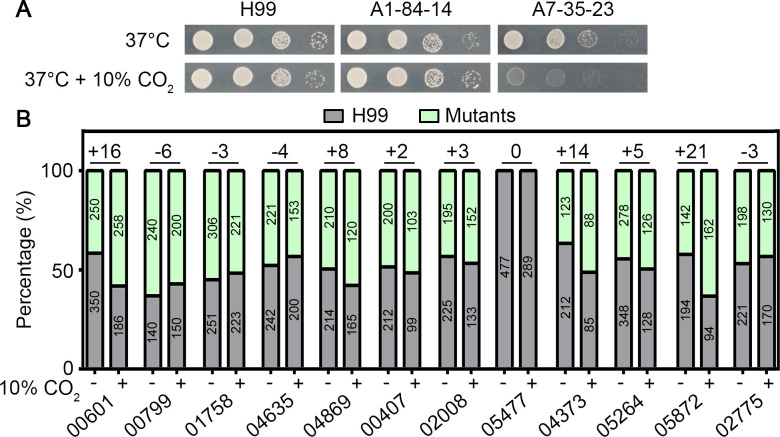
Competition assay reveals that multiple genes encoding secreted proteins selected from the RNA-sequence analysis are involved in cryptococcal growth in high levels of CO_2_. (**A**) The indicated natural isolates were serial diluted, spotted on the YNB medium, and incubated at 37°C in ambient air or in 10% CO_2_ for 2 days. (**B**) The competition assay showed the percentage of the indicated mutants versus the H99 strain of the coculture after incubated in ambient air or 10% CO_2_. The difference in fitness (percentage of the mutant in the coculture) caused by CO_2_ for each mutant was calculated and listed on the top. The number of CFUs for each strain measured in the assay was listed inside the bars.

As an environmental opportunistic pathogen, *C. neoformans* has to be able to adapt to host physiological conditions in order to cause diseases (2, 25). One dramatic difference between its natural niche and mammalian hosts is the level of CO_2_: 0.04% CO_2_ in ambient air versus 5% or above in mammalian tissues (26–28). We previously discovered a positive association between the ability of *C. neoformans* to grow in 5% CO_2_ and their virulence level in mouse models of cryptococcosis (21). To understand the transcriptome differences between CO_2_-tolerant and CO_2_-sensitive strains, we previously carried out RNA deep sequencing experiments using the reference clinical strain H99, and the environmental isolates A1-84-14 and A7-35-23 in 5% CO_2_ or ambient air (21, 23, 24). H99 and A1-84-14 are both CO_2_ tolerant, while A7-35-23 is highly sensitive to CO_2_ (Fig. 1A).

To decipher the unique transcriptome response in the CO_2_-sensitive strain A7-35-23, we identified 466 genes that were differentially expressed (fold change > 2 and *P* < 0.05) in A7-35-23 (5% CO_2_ vs ambient air) but showed no significant change in the CO_2_-tolerant strains H99 and A1-84-14. We decided to focus on 34 of the 466 genes that encode potentially secretory proteins with a signal peptide, because we recently showed that CO_2_ potentiates antifungal drugs that interfere with membrane (21, 23) (Table S1). Of these 34 genes, 16 genes encode products with annotation of predicted function and 14 genes are covered in the H99 deletion sets generated by Dr. Hiten Madhani’s group. We therefore analyzed the CO_2_ sensitivity of these 14 gene deletion mutants by a spotting assay and a competition assay. The spotting assay showed that the *CNAG_05477*Δ was sensitive at 37°C. However, none of the tested mutants showed any obvious increase in sensitivity to high levels of CO_2_ (Fig. S1). In the more sensitive competition assay where the wild-type H99 and the individual mutants were cocultured in ambient air or in 10% CO_2_, the *CNAG_00601*Δ, *CNAG_04373*Δ, and *CNAG_05872*Δ mutants showed significantly enhanced competitive fitness relative to H99 in high CO_2_ condition relative to the ambient air (Fig. 1B). This result suggests that deletion of these genes in *C. neoformans* enhances CO_2_ tolerance, with deletion of *CNAG_05872* (*MAY1*) showing the most drastic effect.

### *MAY1* overexpression sensitizes *C. neoformans* to high CO_2_ due to acidic pH

**Fig 2:**
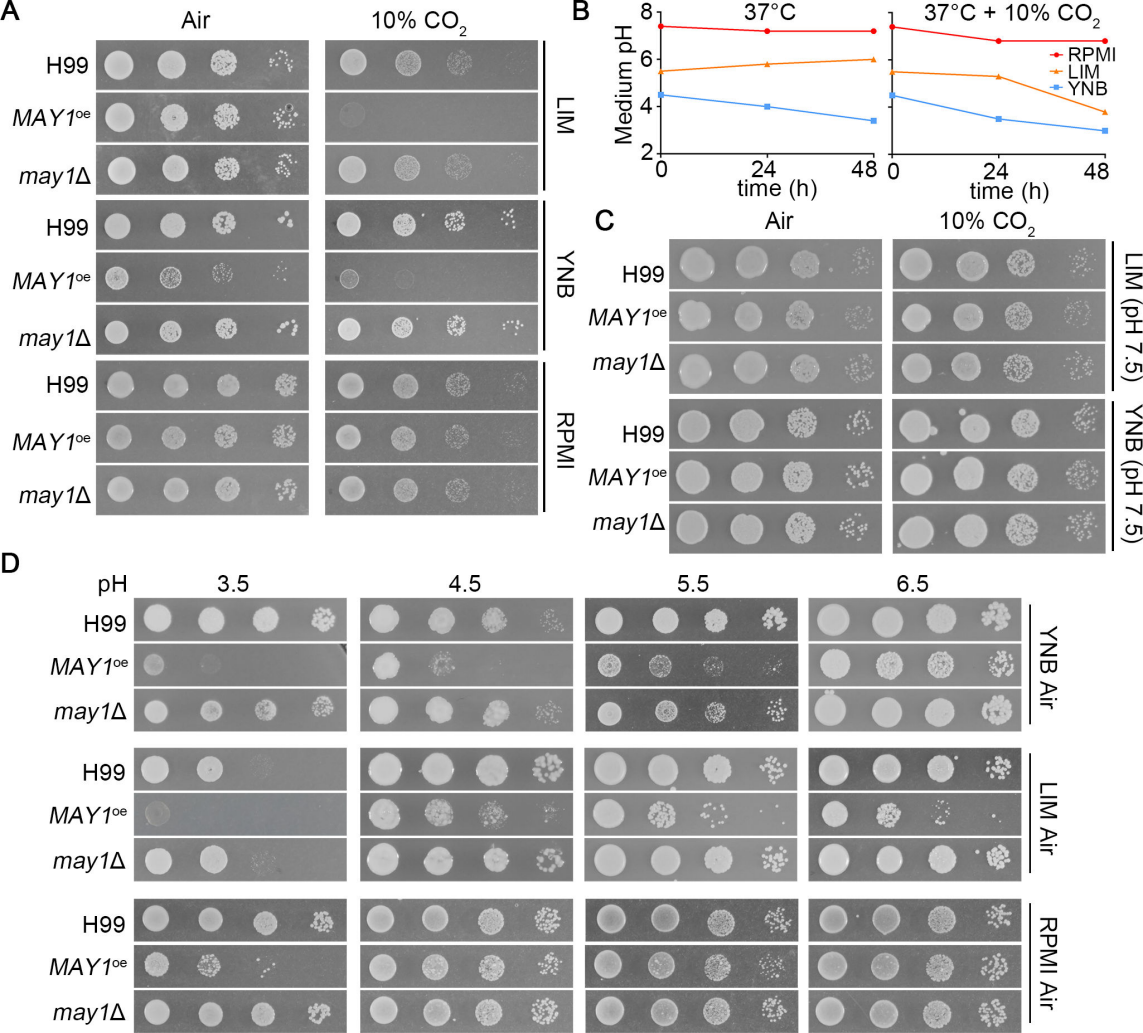
*MAY1*^oe^ dramatically increases cryptococcal sensitivity to CO_2_ due to acidic pH. (**A**) The wild-type H99, the *MAY1*^oe^, and the *may1*Δ strains were serial diluted, spotted onto the YNB, the LIM, and the RPMI media. Cells were incubated at 37°C in ambient air or in 10% CO_2_ for 2 days. (**B**) The reference strain H99 was cultured in LIM, YNB, and RPMI liquid media at 37°C in ambient air or in 10% CO_2_ for 2 days. The medium pH was measured by a pH detector at 0 h, 24 h, and 48 h. (**C**) The indicated strains were serial diluted, spotted onto the buffered YNB and LIM medium (adjusting to pH 7 by adding 50 mM MOPS), and incubated at 37°C in ambient air or in 10% CO_2_ for 2 days. (**D**) The indicated strains were serial diluted, spotted on the YNB, LIM, and RPMI media (adjusting to different pH with 50 mM MOPS), and incubated at 37°C in ambient air for 2 days.

*CNAG_05872* encodes the major aspartyl peptidase May1, which is required for high-density growth under acidic conditions (29, 30). Because deletion of *MAY1* enhances CO_2_ tolerance in the competition assay, we hypothesized that overexpression of *MAY1* will render *Cryptococcus* CO_2_ sensitive. Remarkably, the *MAY1*^oe^ strain barely grew in 10% CO_2_ even in the spotting assay on YNB medium where the defect of the *may1*Δ mutant was not noticeable (Fig. 2A). To determine if the extreme CO_2_-sensitive phenotype of the *MAY1*^oe^ strain is medium dependent, we tested these strains on the mammalian cell culture medium RPMI media and low iron medium (LIM). Interestingly, we found that the *MAY1*^oe^ strain was sensitive to CO_2_ on LIM medium, just like on YNB medium, but not on RPMI medium (Fig. 2A). RPMI medium is buffered to neutral pH, but neither LIM nor YNB medium is buffered. Although we showed previously that CO_2_ sensitivity of the environmental strain A7-35-23 is not pH dependent on the spotting assay (22), we wondered if CO_2_ sensitivity of the *MAY1*^oe^ strain in the originally CO_2_-tolerant H99 background is dependent on pH. To test this hypothesis, we measured medium pH when H99 was cultured in YNB, LIM, or RPMI at 37°C for 0 h, 24 h, or 48 h in ambient air or in 10% CO_2_. The pH of the RPMI medium was relatively stable over time, with a slight drop (7.4 to 7) in 10% CO_2_ (Fig. 2B). The pH of the LIM medium increased slightly over time in ambient air (5.5 to 6) but dropped from 5.5 to 4 in 10% CO_2_ (Fig. 2B). The pH of the YNB medium dropped dramatically from 4.5 to 2.5 in 10% CO_2_ relative to a more modest drop from 4.5 to 3.5 in ambient air (Fig. 2B). Acidification of YNB medium with *Cryptococcus* cultures was previously reported by Upadhya et al. (20). Clarke et al. demonstrated that the purified May1 enzyme is most active at acidic pH (1.9 to 4.0) and the *may1*Δ mutant showed growth defect at acidic pH (31). We noticed a modest growth defect at acidic YNB agar medium of the *may1*Δ mutant, although much drastic growth reduction was observed in the *MAY1*^oe^ strain (Fig. S2A). In liquid YNB media, growth of both *MAY1*^oe^ and *may1*Δ was impaired when the pH dropped (Fig. S2B). Therefore, we hypothesized that the growth defect of the *MAY1*^oe^ strain in 10% CO_2_ on unbuffered YNB and LIM media was due to lowered pH. Indeed, buffering the YNB and LIM media to pH 7.5 with 3-(N-morpholino)propanesulfonic acid (MOPS) abolished the growth defect of the *MAY1*^oe^ strain in 10% CO_2_ (Fig. 2C). Conversely, reducing medium pH caused growth defect of *MAY1*^oe^ regardless of the medium type (Fig. 2D), although the growth defect was more pronounced on LIM or YNB than RPMI medium. This suggests that the more severe growth defects of *MAY1*^oe^ on YNB or LIM medium are influenced by additional factors besides acidic pH.

### Osmotic supplements rescue the growth defect of the *MAY1*^oe^ strain at acidic pH

**Fig 3:**
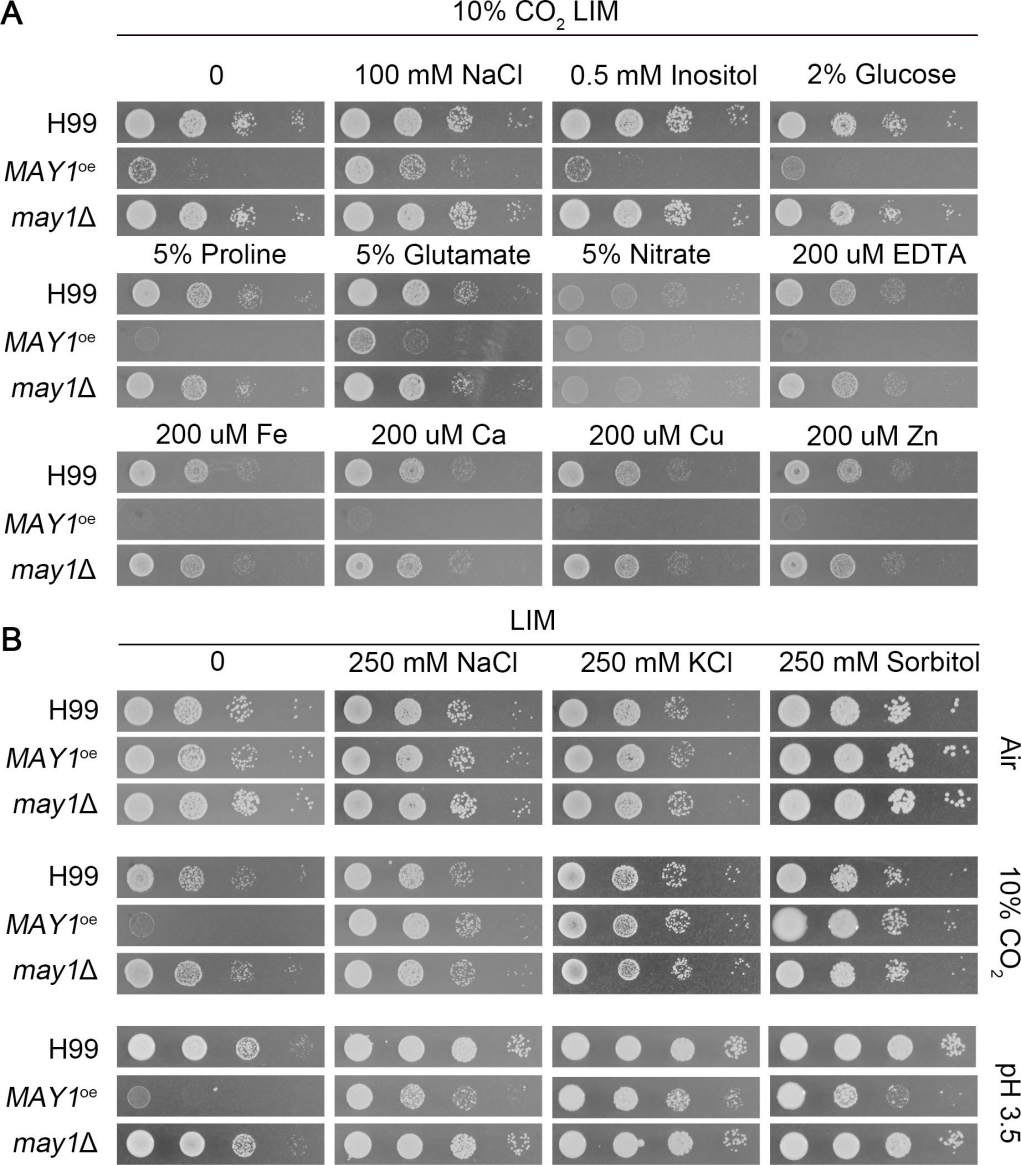
Osmotic supplements rescue the growth defect of the *MAY1*^oe^ strain in acidic pH. (**A**) The wild-type H99, the *MAY1*^oe^, and the *may1*Δ strains were serial diluted, spotted onto the LIM medium with the indicated supplements, and incubated at 37°C in 10% CO_2_ for 2 days. (**B**) The wild-type H99, the *MAY1*^oe^, and the *may1*Δ strains were spotted onto unbuffered LIM medium with or without supplement with 250 mM NaCl, KCl, or sorbitol and incubated at 37°C in ambient air or in 10% CO_2_ for 2 days. The same strains were also spotted onto the LIM medium buffered at pH 3.5 with or without supplement with 250 mM NaCl, KCl, or sorbitol and incubated at 37°C in ambient air for 2 days.

To explore factors other than pH that might have impacted the growth of the *MAY1*^oe^ strain, we separately added the nitrogen source (amino acid glutamate, nitrate), metal ions (iron, calcium, copper, and zinc), the metal chelator EDTA, inositol, and salts (Na^+^) to LIM medium to reduce the difference between LIM and RPMI media. The addition of NaCl (100 mM), but not the other components, partially rescued the growth defect of the *MAY1*^oe^ strain in 10% CO_2_ (Fig. 3A). NaCl at a higher concentration of 250 mM completely rescued the growth defect of the *MAY1*^oe^ strain in 10% CO_2_ (Fig. 3B). Given that NaCl supplementation is commonly used to balance osmolarity, we tested the effect of supplementation by KCl and sorbitol. Supplementation of KCl and sorbitol at 250 mM similarly rescued the growth defect of the *MAY1*^oe^ strain in 10% CO_2_ (Fig. 3B). As we showed earlier that *MAY1* overexpression caused cryptococcal CO_2_ sensitivity due to acidic pH, we tested whether these osmotic supplements could also rescue the growth defect of the *MAY1*^oe^ in acidic pH. As expected, the addition of NaCl, KCl, and sorbitol rescued the growth defect of the *MAY1*^oe^ strain at pH 3.5 (Fig. 3B). These results indicate that osmotic supplements can rescue the growth defect of the *MAY1*^oe^ strain in acidic environments.

### Chitosan deficiency in *MAY1*^oe^ cells is associated with altered morphology and impaired cell wall integrity

**Fig 4:**
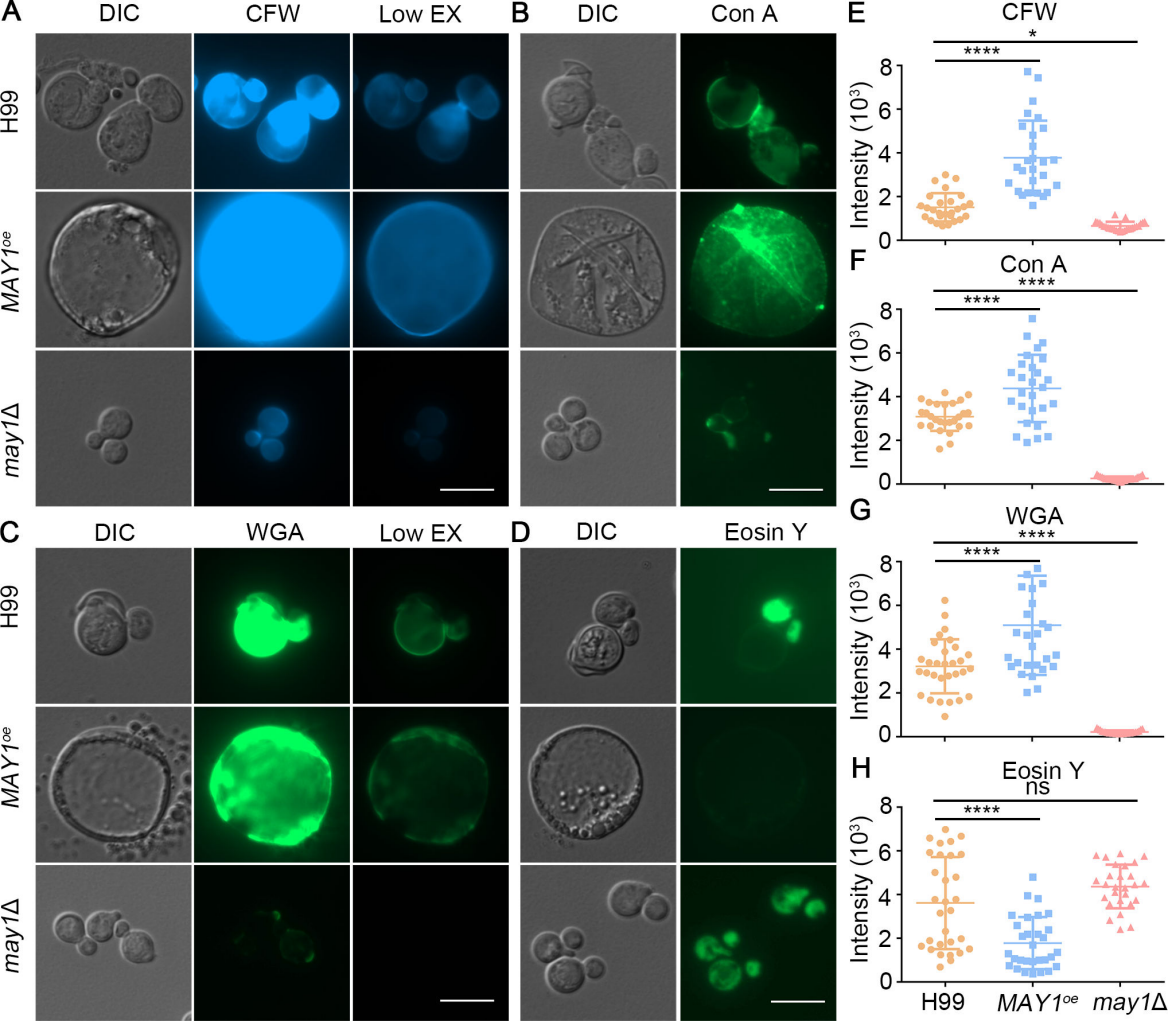
May1 activities enlarge cell size and alter cell wall compositions. Cells of the wild-type H99, the *MAY1*^oe^, and the *may1*Δ strains were grown in liquid YNB medium for 48 h and then stained with calcofluor white (CFW) (**A**), concanavalin A (Con A) (**B**), wheat germ agglutinin (WGA) (**C**), and eosin Y (**D**). (**E–H**) Quantification of the fluorescence intensity in the different strains as detailed in the method section using Zeiss ZEN 3.0 software. Statistical significance was determined using a one-way analysis of variance (ANOVA) statistical analysis. ns, not significant; *P < 0.05 and ****P < 0.0001. Scale bar, 10 µm.

Osmotic supplements such as NaCl, KCl, and sorbitol are known to rescue growth of mutants with a compromised cell wall (32). Upadhya et al. observed that *C. neoformans* grown in unbuffered YNB medium exhibited altered cell morphology and cell wall composition due to acidic pH (20). As pH 1.9–4.0 is the optimal range for May1 activity (31), we hypothesized that May1 may be responsible for the changes in cryptococcal cell morphology and cell wall composition in acidic pH. If this hypothesis was true, then, we predicted that overexpression of *MAY1* would further exacerbate this phenotype while deletion of *MAY1* would mitigate the defect. To test this hypothesis, we cultured H99, the *MAY1*^oe^, and the *may1*Δ strains in liquid YNB medium. Indeed, H99 grown in YNB showed enlarged cells (median 9 µm in diameter compared with 4 µm in diameter in YPD) (Fig. 4A), consistent with the report by Upadhya et al (20). Remarkably, the median diameter of *MAY1*^oe^ cells was 22 µm, while *may1*Δ cells maintained the normal cell size (median: 4 µm in diameter) and morphology (Fig. 4A), suggesting that May1 alters cell morphology of *C. neoformans* grown in YNB medium.

The cell wall of *C. neoformans* is mainly composed of polysaccharides such as glucans, chitin, and chitosan, with mannoproteins being the minor components (12, 33). Upadhya et al. reported enlarged H99 cells grown in YNB medium with altered cell wall composition and organization (20). They found that those enlarged cells have increased levels of chitin, mannoproteins, and β-1,3-glucan and reduced levels of chitosan based on staining using specific dyes (20). To further investigate our hypothesis that May1 activity causes cell wall alterations in acidic pH, we stained cells of H99, *may1*Δ, and *MAY1*^oe^ strains cultured in YNB medium for chitin (CFW), chitosan (eosin Y), chitooligomers (WGA), and mannoproteins (Con A). Indeed, we observed increased staining by CFW (250%), WGA (159%), and Con A (139%) in the *MAY1*^oe^ strain relative to the H99 control (Fig. 4A through C and Fig. 4E through G; Fig. S3A). Conversely, we observed reduced staining of CFW (45%), WGA (4%), and Con A (2%) in the *may1*Δ mutant (Fig. 4A through C and Fig. 4E through G; Fig. S3A). In contrast to CFW, WGA, and Con A staining, the *MAY1*^oe^ strain showed reduced staining by eosin Y (48%), while the *may1*Δ mutant showed enhanced staining (123%) (Fig. 4D and H). We noticed larger variation in eosin Y staining in the wild-type H99 cells compared with the *may1*Δ mutant (Fig. 4H). These data indicate that chitosan deficiency is further exacerbated in *MAY1*^oe^ cells.

Consistent with our earlier observation that osmotic supplements rescued the growth defect of the *MAY1*^oe^ strain in acidic pH, NaCl supplement rescued the cell size enlargement defect of the *MAY1*^oe^ strain cultured in YNB medium (Fig. S3B). As predicted, when the *MAY1*^oe^ strain was cultured in YNB medium buffered to neutral pH, the cell size was similar to that of H99 (Fig. S3B).

### May1 remodels cryptococcal cell wall through influencing Chs3

In *C. neoformans*, chitin produced by the chitin synthase Chs3 is deacetylated by the three chitin deacetylases Cda1-3 to form chitosan (17–19). Because overexpression of *MAY1* caused increased chitin content and decreased chitosan content, we postulated that overexpression of chitin deacetylase genes in the *MAY1*^oe^ strain may help convert the excess chitin to chitosan and consequently rescue its growth defect in acidic pH. To test our hypothesis, we overexpressed *CDA1*, *CDA2*, and *CDA3* individually in the *MAY1*^oe^ strain and confirmed their overexpression by real-time quantitative PCR (Fig. S4A). Contrary to our prediction, none of the three *CDA* genes rescued the CO_2_-sensitive phenotype of the *MAY1*^oe^ strain when overexpressed (Fig. S4B). This result suggests that chitosan deficiency of the *MAY1*^oe^ strain in CO_2_ cannot be compensated by overproduction of these chitin deacetylases.

**Fig 5:**
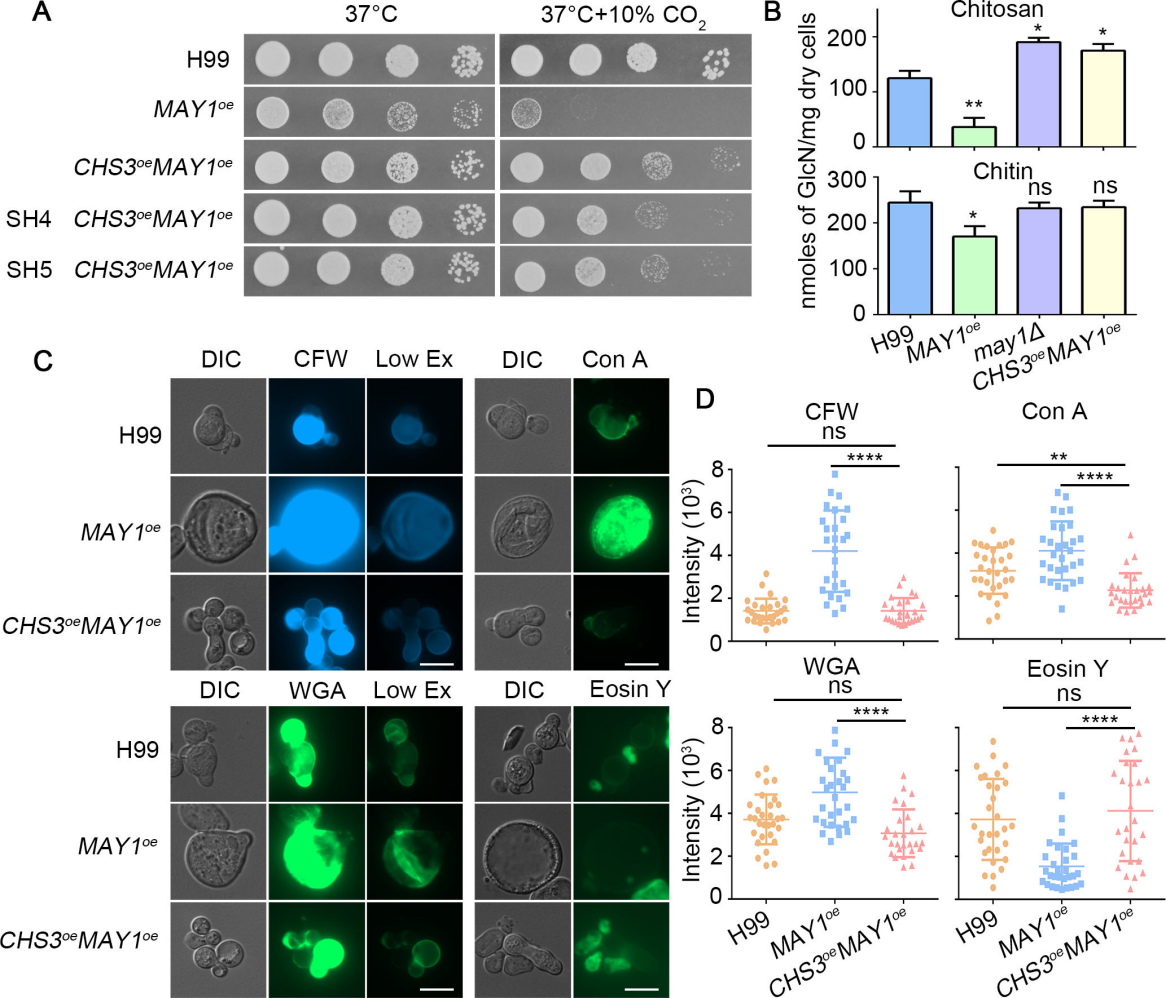
*CHS3*^oe^ rescues the chitosan deficiency of the *MAY1*^oe^ strain. (A) The indicated strains were serially diluted, spotted onto the YNB, and incubated at 37°C in ambient air or in 10% CO_2_ for 2 days. (B) The amount of chitin and chitosan was measured using the MBTH method after the indicated cells were grown in the YNB medium for 2 days at 37°C. (C) The indicated cells were grown in liquid YNB medium for 48 h and then stained with CFW, Con A, WGA, and eosin Y. Representative images are shown here. (D) Quantification of the fluorescence intensity of the different strains as detailed in the method section.

Because chitosan is converted from chitin specifically synthesized by Chs3 (19), we then wondered whether the lack of chitin specifically synthesized by Chs3 caused the chitosan deficiency in the *MAY1*^oe^ strain. To test this idea, we overexpressed *CHS3* in a *MAY1*^oe^ strain where the *CHS3*^oe^ construct was integrated into the safe haven *SH2* site and the *MAY1*^oe^ construct was integrated ectopically. Remarkably, the growth defect of the *MAY1*^oe^ strain in CO_2_ condition was largely rescued by the overexpression of *CHS3* (third row in Fig. 5A). To ensure the impact of *CHS3*^oe^ we observed was not due to a position effect of the *MAY1*^oe^ construct integration, we integrated the *MAY1*^oe^ construct at one of the two unlinked genetic sites we identified and named as “safe haven” 4 and 5 (*SH4* and *SH5* in Fig. S5A). We confirmed that the integration of *MAY1*^oe^ at *SH4* or *SH5* did not alter the expression of genes bordering the *SH4* and *SH5* sites (Fig. S5B). The *MAY1* transcript level in the *CHS3*^oe^*MAY1*^oe^ strains with *MAY1*^oe^ integrated at the *SH4* or the *SH5* site was comparable (Fig. S5C). When we tested these double-overexpression strains, we found that *CHS3*^oe^ largely restored the growth defect of the *MAY1*^oe^ strain in CO_2_ regardless whether the construct was integrated at the *SH4* site, the *SH5* site, or ectopically (Fig. 5A).

Since the overexpression of *CHS3* rescued the growth defect of the *MAY1*^oe^ strain in CO_2_, we speculated that chitosan deficiency of *MAY1*^oe^ might be restored by *CHS3* overexpression. To better measure the amount of chitosan in the *MAY1*^oe^ and the *CHS3*^oe^*MAY1*^oe^ strains, we biochemically quantified the levels of chitosan subunit glucosamine from their cell walls (19, 34). Consistent with the chitosan staining, the *MAY1*^oe^ strain showed decreased chitosan content (29% relative to H99), while the *may1*Δ mutant showed increased chitosan content (153%) (Fig. 5B). Moreover, *CHS3*^oe^*MAY1*^oe^ showed 386% increase in chitosan content compared with that of *MAY1*^oe^, suggesting that overexpression of *CHS3* indeed rescued the chitosan deficiency of *MAY1*^oe^. As chitosan deficiency alters cell wall composition and enlarges cell size (19), we speculated that overexpression of *CHS3* should also rescue these associated phenotypes as well. Indeed, the cell size of the double overexpression *CHS3*^oe^*MAY1*^oe^ strain (median: 10 µm in diameter) was similar to that of H99 (median: 9 µm in diameter) (Fig. 5C). Moreover, overexpression of *CHS3* in the *MAY1*^oe^ strain reduced the elevated staining of CFW, Con A, and WGA and raised the decreased staining of eosin Y, making the signals comparable to the wild-type control (Fig. 5D; Fig. S6). These data support the idea that deficiency in chitosan of the *MAY1*^oe^ strain resulted from the lack of chitin synthesized by Chs3. We noted that chitin measurement by CFW staining and biochemical quantification of glucosamine gave conflicting results on the relative abundance of chitin in *MAY1*^oe^ cells: CFW signal was the strongest in *MAY1*^oe^ cells relative to WT H99 and *CHS3*^oe^*MAY1*^oe^, but glucosamine measurement indicated a modestly reduced chitin level in *MAY1*^oe^ cells relative to WT H99 and *CHS3*^oe^*MAY1*^oe^. Upadhya et al. also observed similarly conflicting data on the chitin level by these two measurements, and they postulated that CFW may bind non-specifically to an unknown component in the cell wall (20).

**Fig 6:**
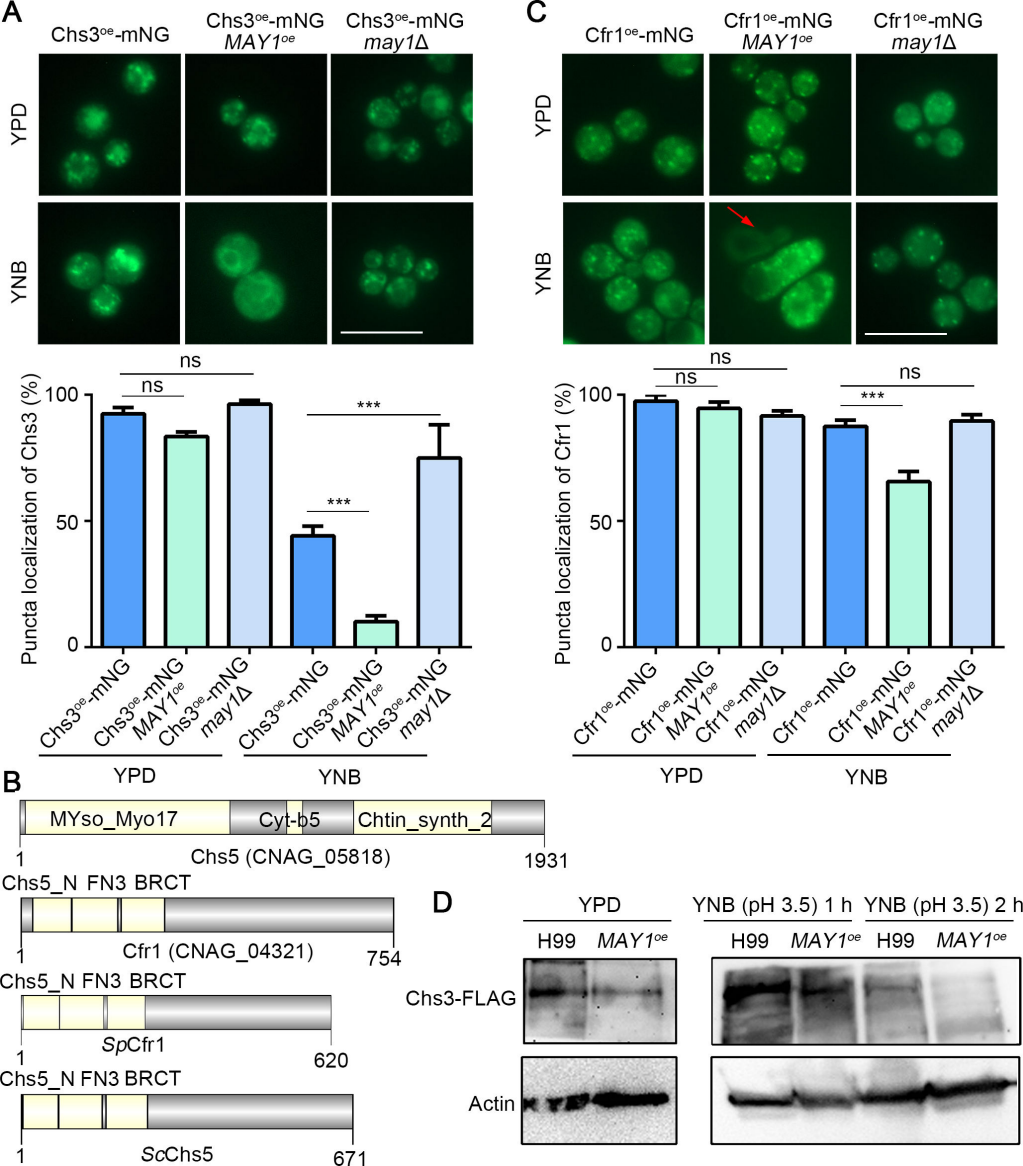
May1 influences the localization of Chs3. (**A**) The indicated strains with P*_GPD1_-CHS3*-mNG were cultured in liquid YPD or YNB medium at 37°C for 2 days. Chs3-mNG puncta of the corresponding strains were quantified as detailed in the Materials and Methods section (*n* = 60). (**B**) Protein diagram of *Sp*Cfr1 in *S. pombe*, *Sc*Chs5 in *S. cerevisiae*, and Cfr1 (CNAG_04321) and Chs5 (CNAG_05818) in *C. neoformans*. (**C**) The Cfr1 fluorescently tagged strains were cultured in liquid YPD or YNB medium at 37°C for 2 days. Cfr1 puncta of the different strains were quantified as detailed in the Materials and Methods section (*n* = 60). (**D**) The indicated strains were cultured in liquid YPD at 37°C for 2 days and then transferred to YNB (pH 3.5) for 1 h or 2 h. Statistical significance was determined using a one-way ANOVA statistical analysis. ns, not significant; ***P < 0.001.

To further explore how May1 influences Chs3, we overexpressed Chs3 fused with mNeonGreen/mNG using a constitutively active promoter of *GPD1* (glycerol-3-phosphate dehydrogenase 1) in H99, the *MAY1*^oe^, the *may1*Δ, and the *chs3*Δ strains. The *CHS3*^oe^-mNG rescued the temperature-sensitive phenotype of the *chs3*Δ mutant at 40°C (Fig. S7A), suggesting that tagged Chs3 is functional. In YPD medium (no acidification-May1 inactive), the vast majority of cells showed puncta localization of Chs3-mNG in all strains: 92% of H99 cells, 83% of *MAY1*^oe^ cells, and 96% of *may1*Δ cells (Fig. 6A; Fig. S7B). In YNB medium (acidified), the population with puncta localization of Chs3-mNG decreased to varied degrees in different strains: 44% of H99 cells, 10% of the *MAY1*^oe^ strain, and 72% of the *may1*Δ mutant (Fig. 6A; Fig. S7B). Thus, disappearance of Chs3 puncta appears to be positively correlated with May1 activity. This phenotype is not an artifact resulting from overexpression driven by a constitutive promoter, as native promoter-driven Chs3-mNG in H99 and the *MAY1*^oe^ strain background gave similar results even though the fluorescence intensity of Chs3-mNG was dimmer (Fig. S7C and D).

In *S. cerevisiae*, *Sc*Chs3 is synthesized in the endoplasmic reticulum, sorted in the trans-Golgi, and trafficked to the plasma membrane by exomer (35–38). To determine the subcellular localization of Chs3 in *C. neoformans*, we fluorescently marked ER and Golgi in the Chs3-mNG strain and found that most of Chs3-mNG puncta localized to Golgi (Fig. S7E) (39), which is similar to *Sc*Chs3. *Sc*Chs5 is important for forming the exomer complex, which is involved in trafficking *Sc*Chs3. As expected, *Sc*Chs5 and *Sc*Chs3 show similar localization patterns (37, 40–42). In *C. neoformans*, Chs5 is actually a chitin synthase (19), and it does not harbor any domains similar to those present in *Sc*Chs5 (Fig. 6B). Here, we used the *Sc*Chs5 protein sequence as a query and found *C. neoformans* Cfr1 encoded by *CNAG_04321* as the best match. The predicted Cfr1 protein possesses similar domains as *Sc*Chs5 in *S. cerevisiae* and *Sp*Cfr1 in *S. pombe* (Fig. 6B). When Cfr1 fused with fluorescent protein mNG showed puncta localization in the vast majority of cells in all strains in the YPD medium: 97% of H99 cells, 94% of *MAY1*^oe^, and 92% of *may1*Δ cells (Fig. 6C; Fig. S7E). This Cfr1 localization in YPD is similar to that of Chs3. In YNB medium, however, majority of cells retained their Cfr1 punctate localization in all strains: 87% of H99 cells, 65% of *MAY1*^oe^, and 89% of *may1*Δ cells (Fig. 6C; Fig. S7E). This pattern differs from Chs3 where a more drastic reduction in puncta was observed in YNB media. Nonetheless, the minority of the *MAY1*^oe^ cells that lost puncta localization of Cfr1-mNG also showed decreased fluorescence intensity (Fig. 6C; Fig. S7E). Taken together, these results indicated that May1 has a stronger effect on Chs3 relative to Cfr1. As May1 is an aspartyl peptidase, we speculated that disappearance of Chs3 puncta could result from degradation of Chs3 by May1 in acidic pH. To test this hypothesis, we constructed the Chs3^oe^-FLAG tag strain in both H99 and the *MAY1*^oe^ strain background and monitored the Chs3 level by western blot. We detected a slightly stronger Chs3 signal in H99 than in the *MAY1*^oe^ strain background (Fig. 6D). When we shifted the cultures from YPD media to YNB media (pH 3.5), the level of Chs3 in the *MAY1*^oe^ background became much lower than that in H99 even after just 1 h after the shift. The Chs3 level was dramatically reduced in both backgrounds by 2 h after the shift, and Chs3 in the *MAY1*^oe^ strain became undetectable. These results indicate that May1 is involved in Chs3 degradation.

### Cryptococcal cells with *MAY1* overexpression induce hyper-inflammatory response

**Fig 7:**
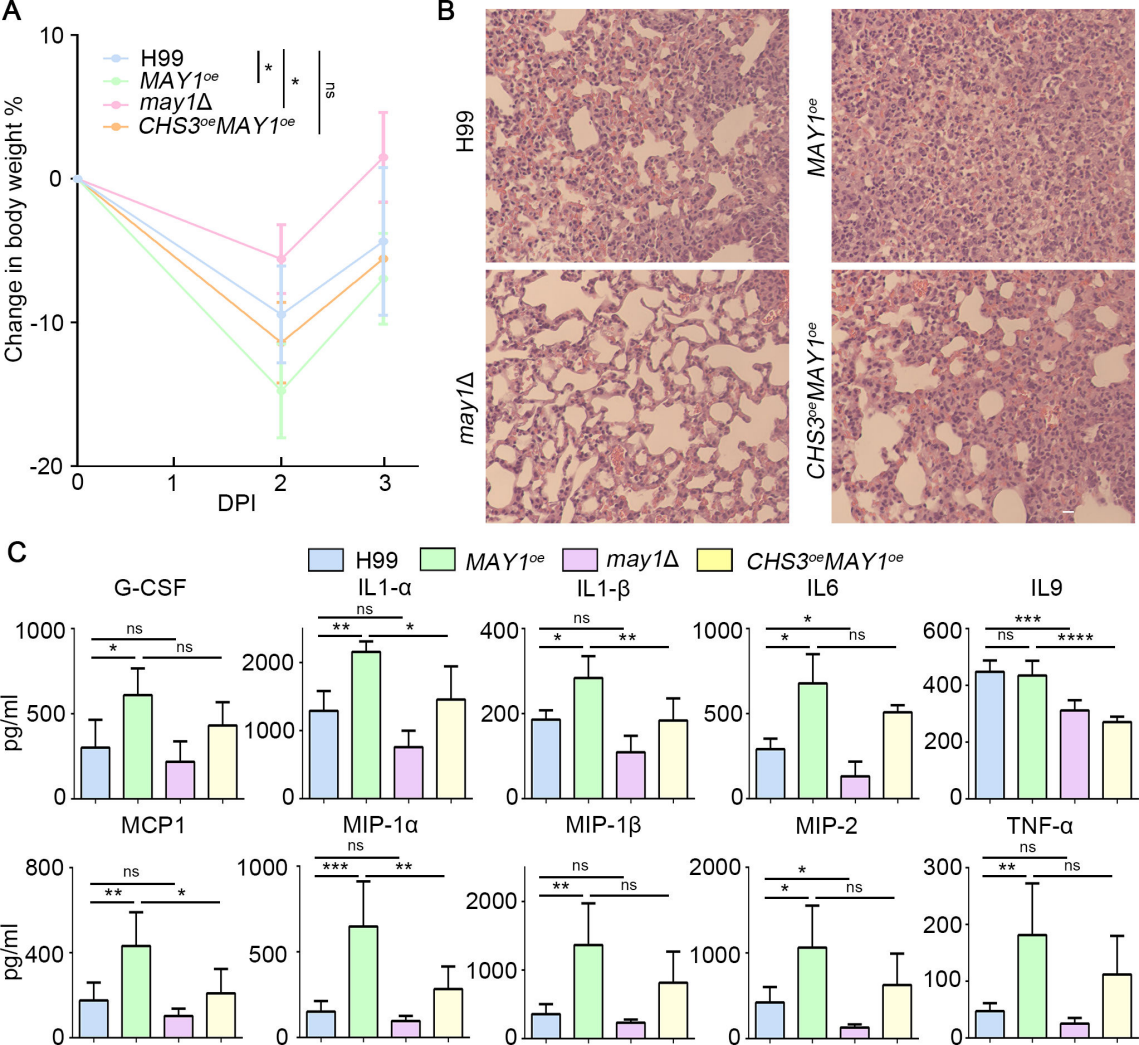
May1 activities in *Cryptococcus* correlate with host hyper-inflammatory response. (**A**) Body weight changes in the mice inoculated with HK cells of the indicated strains. (**B**) Hematoxylin and eosin (H&E) staining of the lungs of mice inoculated with HK cells of the indicated strains at DPI 3. (**C**) Cytokines and chemokines recovered from the supernatant of homogenized lungs of the indicated groups (*n* = 5/group). Statistical significance was determined using a one-way ANOVA statistical analysis. ns, not significant; *P < 0.05, **P < 0.01, ***P < 0.001, and ****P < 0.0001.

Upadhya, Hole, and their colleagues found that heat-killed cryptococcal cells lacking chitosan induced a proinflammatory host response (20, 43, 44). Since the chitosan content is reduced in the *MAY1*^oe^ strain, we hypothesized that the *MAY1*^oe^ strain cultured in acidic media may cause an exacerbated inflammatory response, while the *MAY1* deletion cells may elicit a subdued response from the host compared with the wild-type control. To this end, we inoculated CBA/J mice with heat-killed cells of the H99, *may1*Δ, *MAY1*^oe^, and *CHS3*^oe^*MAY1*^oe^ strains grown in YNB medium. We found that all groups of mice lost body weight at day 2 post inoculation (DPI 2) and then recovered at DPI 3 (Fig. 7A). Mice exposed to HK *MAY1*^oe^ cells lost most of their body weight compared with other groups (Fig. 7A). The degree of weight loss in mice exposed to HK *CHS3*^oe^*MAY1*^oe^ cells was similar to that in mice inoculated with HK H99 cells (Fig. 7A), consistent with the idea that overexpression of *CHS3* largely restores the defect of the *MAY1*^oe^ strain. As we predicted, mice exposed to HK *may1*Δ cells lost the least amount of body weight.

The temporary loss of body weight may reflect the inflammatory responses of the host. Indeed, histological analysis of the lung sections stained with H&E revealed the most intense inflammatory cell infiltration in mice exposed to HK *MAY1*^oe^ cells, followed by the *CHS3*^oe^*MAY1*^oe^ group, the H99 group, and then the *may1*Δ group (Fig. 7B; Fig. S8). To quantify the differences of inflammatory response between the groups, we examined the levels of 25 murine cytokines/chemokines from lung homogenates using Luminex multiplex assays. As expected, we found much higher levels of cytokines/chemokines that are associated with inflammation—interleukins (IL1-α, IL1-β, and IL6), monocyte chemoattractant protein (MCP1), macrophage inflammatory proteins (MIP-1α, MIP-1β), and tumor necrosis factor alpha (TNF-α)—in mice exposed to HK *MAY1*^oe^ cells, followed by HK H99 and HK *CHS3*^oe^*MAY1*^oe^ cells (Fig. 7C) (45, 46). Again, mice exposed to HK *may1*Δ cells generally produced the lowest levels of these cytokines/chemokines (Fig. 7C). Collectively, these results demonstrated that May1 activity correlates with the degree of host inflammatory response.

## DISCUSSION

Aspartyl proteases are endopeptidases with conserved Asp-Gly-Thr amino acids at their active site (47, 48). These enzymes become active at acidic pH (pH 1.9 to 4.0) (29, 48, 49). In phytopathogens, aspartyl proteases degrade proteins in the host plant cell wall during infection, which facilitates host colonization and penetration, and released amino acids also serve as the principal source of nitrogen to the pathogen (50–52). In human fungal pathogens, proteolytic activities of aspartyl proteases in *C. albicans*, *A. fumigatus*, and *C. neoformans* also aid these pathogens in the colonization and penetration of host tissues (31, 49, 53–56).

As reported by Upadhya et al., *C. neoformans* acidifies YNB medium at 30°C and its cell wall shows greatly reduced levels of chitosan but highly increased levels of chitin, β-1,3-glucan, and mannans (20). However, how the acidic environment causes chitosan deficiency in this fungus was unclear. In this work, we recapitulated the same phenotype of wild-type H99 grown in YNB medium at 37°C and found that higher temperature accelerated this process. We found that May1, an aspartyl peptidase initially discovered by Clarke et al. in 2016 (31), is likely the major culprit responsible for this phenomenon. Clarke and colleagues found that May1 activity is optimal at an acidic pH between 3.5 and 4.5 and the deletion of *MAY1* causes a modest growth defect at an acidic environment (31). Here, we recapitulated the same modest phenotype of the *may1*Δ mutant and surprisingly found that overexpression of *MAY1* causes much severe growth defect in an acidic environment. We discovered that overexpression of *MAY1* exacerbates chitosan deficiency while the deletion of *MAY1* nearly abolishes the phenotype. Thus, *C. neoformans* acidifies YNB medium, which activates May1, causing remodeling of the cryptococcal cell wall. *S. cerevisiae* is known to respond to low external pH by remodeling the cell wall (57). Thus, we speculated the growth defect of *may1*Δ and more so of the *MAY1*^oe^ strain in the acidic environment resulted from defects in cell wall remodeling. The defect of the *MAY1*^oe^ strain in acidic pH is largely due to the lower chitosan level, and the specifics of cell wall defect in the *may1*Δ mutant are not yet clear.

In *C. neoformans*, Chs3 synthesizes the majority of chitin used to be converted to chitosan by chitin deacetylases (17–19). Therefore, reducing the function of either Chs3 or the chitin deacetylases by May1 could have resulted in a reduced chitosan level at acidic pH. We found that overexpression of *CHS3*, but not *CDA1-3*, largely rescued chitosan deficiency and associated phenotypes in the *MAY1*^oe^ strain. This suggests that chitosan deficiency of the *MAY1*^oe^ strain results from an insufficient supply of chitin produced by Chs3. As reported, palmitoylated Chs3 by Pfa4 in *C. neoformans* is localized in the internal compartments and plasma membrane (58). Here, we found that May1 causes disappearance of Chs3 and Cfr1 puncta in Golgi, with a larger impact on Chs3. Accordingly, relative to *CHS3* overexpression, *CFR1* overexpression more effectively rescues the growth defect of *MAY1*^oe^ in high CO_2_ conditions (Fig. S7G). It is possible that Cfr1 and Chs3 may be packaged in the same vesicles as May1, and abundant Cfr1 may shield Chs3 from being cleaved by May1. Although mature secretory granules or multivesicular body (pH 5–6) are usually only weakly acidic (59, 60) and are beyond the optimal pH range of May1 activity, May1 still keeps 20% activity in this pH range. Given that yeast cells reduce cytosolic pH in response to low external pH (61), it is possible that the pH of vesicles may be lower in an acidic environment, which will then augment the activity of May1.

Chitosan plays a critical role in modulating host immune response to *C. neoformans* (43, 44, 62). Infection with the chitosan-deficient *cda1-3*Δ strain led to a Th-1-type adaptive protective response (43). Here, we noted that overexpression of *MAY1* at acidic pH reduced the chitosan contents in the cell wall and increased exposure of surface PAMPs. However, heat-killed *MAY1*^oe^ cells also induced a hyper-inflammatory response. Inconsistent with previous studies, chitosan-deficient *chs3*Δ strain or the wild-type strain grown in unbuffered YNB medium (acidic condition) cause an uncontrolled hyper-inflammatory response in mice (20, 44). Thus, it would be tricky to balance the potential beneficial and damaging effects of May1 overexpression. By contrast, heat-killed *may1*Δ cells dampen the inflammatory response compared with heat-killed H99 cells. Because heat-killed H99 cells grown in YNB-buffered medium provides protective immunity without causing damaging inflammatory response (20), it is possible that deletion of *MAY1* in some of the current or future vaccination strains (63–68) may reduce tissue-damaging inflammatory responses.

## MATERIALS AND METHODS

### Strains and culture conditions

*Cryptococcus neoformans* strains and plasmids used in this study are listed in the Table S2. All strains were stored as 15% glycerol stocks at −80°C and cultured in YPD (1% yeast extract, 2% peptone, 2% dextrose, and 2% agar) at 30°C unless stated otherwise. YNB (0.67% yeast nitrogen base with no amino acids, 2% dextrose, and 2% agar), RPMI (catalog no. SH30011.04, Cytiva), LIM were used to test the phenotypes (69).

### Competition assay

All mutants and the wild-type H99 were cultured individually in liquid YPD medium at 30°C with shaking at 220 rpm for 16 h. Cells were collected and washed with sterile ddH_2_O and then adjusted to the same cell density (optical density at 600 nm = 0.1). Each mutant was mixed with H99 in a 1:1 ratio, and then, the mixture was spotted onto the YNB medium. The co-cultures were incubated at 37°C for 48 h in either ambient air or in 10% CO_2_. The colonies were collected and then serially diluted. Aliquots of the dilutions (100 µL) were spread onto YPD and YPD with nourseothricin and incubated at 30°C for 2 days so that the colonies became visible for counting. Because all mutants carry the nourseothricin resistance marker NAT, the CFUs on YPD medium were the total counts of H99 + mutant (Nt = N total) whereas CFUs on YPD + NAT only measured the mutant counts (Nm = N mutant). The fitness index of the mutant was calculated as Fm = (Nm/Nt) * 100 with 50% meaning the mutant was as fit as H99 under that culture condition. The difference in fitness caused by CO_2_ for each mutant was calculated as Fm (CO_2_) – Fm (ambient air) (the numbers listed in Fig. 1C). CO_2_ levels were controlled by a VWR CO_2_ incubator or by a Pro-CO_2_ controller (Biospherix, Lacona, NY, USA).

### Differential expression analysis

The fragments per kilobase of transcript per million mapped reads (FPKM) values were generated from the published RNA-seq data (GEO: GSE260932) using Trim_Galore (0.6.5), STAR (2.7.1a), and Cufflinks (2.2.1). Differential gene expression analysis was performed using DESeq2.

### Gene manipulation

For gene overexpression or fluorescence labeling driven by a constitutively active promoter, the entire open reading frame was amplified, digested with *Fse*I and *Pac*I, and then cloned into vectors harboring the *TEF1* or *GPD1* promoter with a mNeoGreen tag as we described previously (70, 71). All strains from H99 deletion set generated by Dr. Hiten Madhani’s group were purchased from Fungal Genetics Stock Center, and the gene deletion of the mutants used here was confirmed by diagnostic PCR. The *CNAG_02008*Δ and *CNAG_02775*Δ from the deletion set were found to be incorrect. For the generation of gene deletion strains, a deletion construct that contains approximately 1 kb of flanking sequences and drug marker was constructed by overlap extension PCR. The constructs were introduced into the recipient strains by TRACE (71, 72). All the primers used in this study were listed in Table S3.

### Phenotypic assays

The indicated strains were cultured in liquid YPD medium at 30°C with shaking at 220 rpm for 16 h. Cells were washed with sterile ddH_2_O and adjusted to the same cell density (optical density at 600 nm = 1.0) and then serially diluted. The serial dilutions of each strain (3 µL) were spotted onto various agar media. The medium pH was adjusted by adding 50 mM MOPS. To test the major components that differ between RPMI and LIM media, cells were grown on LIM medium supplemented with 100 µM NaCl, 0.5 mM inositol, 200 µM EDTA, 200 µM FeCl_3_, 200 µM CaCl_2_, 200 µM CuSO_4_, or 200 µM ZnCl_2_.

### Staining of different cell wall-specific molecules

The indicated strains were grown in the YNB medium at 37°C with shaking at 220 rpm for 48 h. Cells were collected and washed with sterile ddH_2_ O and adjusted to the same cell density. For staining, CFW, WGA, Con A, and eosin Y were used at 1 µg/mL, 100 µg/mL, 50 µg/mL, and 25 µg/mL, respectively. The fluorescent images were taken with a AxioCam 506 mono camera hooked to a Zeiss Imager M2 microscope (Zeiss, Oberkochen, Germany). The fluorescence intensity of 30 individual cells from each strain was quantified using ZEN “Histo definition” quantification software. Each cell and its background were selected using the circular selection tool, and the average fluorescence intensity for the selected area was recorded.

### Identification of safe haven sites *SH4* and *SH5*

To integrate the overexpression constructs at loci other than the established *SH2* site, additional safe haven sites need to be identified. We used the same procedures to identify the additional safe heaven sites as we described previously (73–75). Briefly, the genomic sequence and annotation files of *C. neoformans* H99 were downloaded from NCBI (GenBank assembly accession: GCA_000149245.3). We classified the intergenic regions and calculated the sizes of these intergenic regions based on the orientation and positions of two neighboring genes (73–75). BAM (binary alignment map) files and FPKM values were generated using Trim_Galore (0.6.5), STAR (2.7.1a), and Gufflink (2.2.1). BAM files were visualized with Integrative Genomic Viewer (2.6.3) to check the transcript of the intergenic region and neighboring genes. We chose convergent intergenic regions (tail-tail orientation of the two bordering genes) larger than 2 kb with detectable expression of their neighboring genes to exclude the potential heterochromatin regions. We obtained two safe haven candidates that we named *SH4* (*CNAG_02589-SH4-CNAG_02589*) and *SH5* (*CNAG_06526-SH5-CNAG_06527*) as indicated in Fig. S5A. Integration of DNA in these two regions did not appear to affect the expression of the neighboring genes (Fig. S5B).

### Cell wall chitin/chitosan measurement

Cell wall chitin and chitosan levels were measured by a modified MBTH (3-methyl- benzothiazolinone hydrazone hydrochloride) method (19, 34, 76). Briefly, the indicated strains (optical density at 600 nm = 0.1) were grown in YNB medium at 37°C with shaking at 220 rpm for 48 h. Cells were harvested, washed, lyophilized, and weighed. For each sample, lyophilized cells of the same dry weight (10 mg) were resuspended in 10 mL 6% KOH and incubated at 80°C for 90 min. Cells were collected by centrifugation, washed with H_2_O, and then resuspended in H_2_O to a concentration of 10 mg/mL. The cell suspension was then sonicated for 2 min to homogenize the samples using the VWR ultrasonic baths. For each sample, two duplicated cell suspensions were prepared (one for chitosan only and the other for chitin + chitosan). D-glucosamine solutions with concentrations ranging from 5 µM to 50 µM were used as the standards for chitosan. 100 µL cell suspensions or standard samples were mixed with 100 µL 1M HCl and mixed by vortexing. The tubes for assaying chitin + chitosan were incubated at 100°C for 2 h, while tubes for measuring chitosan alone were incubated at 22°C. Chitin in hot HCl will be deacetylated to become chitosan. Next, all samples were deaminated as follows: 400 µL of 2.5% sodium nitrite was added to the tubes, vortexed, and then left at 22°C for 15 min in a fume hood. Then, 200 µL of 12.5% ammonium sulfamate was slowly added to the tubes, and then, the mixture was incubated at 22°C for 5 min. 200 µL 0.25% MBTH was added to each sample, mixed by vortexing, and incubated at 37°C for 30 min. Next, 200 µL 0.5% ferric chloride was added and the samples were then incubated at 37°C for another 5 min. The samples were cooled down and spun for 2 min. 200 µL supernatant was added to a 96-well plate and measured at 650 nm using BioTeck Epoch 2 Microplate Spectrophotometer.

### Fluorescence microscopy

The indicated strains were cultured in liquid YPD or YNB media at 37°C for 2 days and washed three times with sterile ddH_2_O. Images were acquired by using a Zeiss Imager M2 microscope (Zeiss, Oberkochen, Germany) with an Axio-Cam MRm camera and processed with Zen pro software. The percentage of Chs3 or Cfr1 puncta localization was calculated based on the measurement of 60 individual cells from each strain examined using a 63× objective.

### Western bolt

Proteins were extracted from the indicated strains following a previously described method (77). Aliquots of proteins were separated on 12% SDS-PAGE gels and then transferred to polyvinylidene difluoride membrane for analysis using anti-FLAG (Sigma) and anti-actin (Thermo Fisher) antibody.

### Histology analysis

Female CBA/J mice of 8 to 10 weeks old were purchased from the Jackson laboratory (Bar Harbor, ME). Cryptococcal strains were inoculated in 3 mL YNB medium (initial inoculum optical density at 600 nm [OD_600_] = 0.1) and cultured at 37°C with shaking at 220 rpm for 2 days. Cells were washed with sterile saline three times and adjusted to a final concentration of 2 × 10^5^ cells/mL. For heat inactivation of cells, the cell suspension was heated at 65°C for 20 min. Mice were sedated with ketamine and xylazine via intraperitoneal injection and then inoculated intranasally with 50 µL heat-killed fungal suspension using the same procedures as we described previously (66, 67, 78). The change of body weight of each animal was calculated as follows: (weight on day X − weight before infection)/weight before infection × 100%. The animals were euthanized on day 3 post inoculation, and their lungs were collected. These collected organs were fixed in 10% formalin, embedded in paraffin, sliced into 5-μm-thick sections, and processed with H&E staining at a veterinary diagnostic laboratory at the College of Veterinary Medicine in the University of Georgia.

### Pulmonary cytokine measurement

The lungs of euthanized mice inoculated with the heat-killed cryptococcal cells were dissected and homogenized in 1 mL cold PBS with beads for 2 min. The mixtures were centrifuged at 500 × *g* for 5 min, and the collected supernatant was then centrifuged at 5,000 *× g* for 5 min. The supernatant collected was then used for measurement of the cytokines and chemokines using the MILLIPLEX Mouse Cytokine/Chemokine Magnetic Bead Panel (MCYTOMAG-70K, MilliporeSigma) according to the instruction of the manufacturer. The signals were detected using Luminex MAGPIX at the CVM cytometry core facility of the University of Georgia (79).

## Data Availability

All data supporting this study are presented herein, and the reported fungal strains generated for this study are available on request.

## SUPPLEMENTAL MATERIAL

Supplemental material mbio.00920-24-s0001.pdf Figures S1-S8 and Tables S1-S2.

ASM does not own the copyrights to Supplemental Material that may be linked to, or accessed through, an article. The authors have granted ASM a non-exclusive, world-wide license to publish the Supplemental Material files. Please contact the corresponding author directly for reuse.
