# Supplementary material for: Aspartyl peptidase May1 induces host inflammatory response by altering cell wall composition in the fungal pathogen Cryptococcus neoformans
Source: mBio. 2024 May 14;15(6):e00920-24. doi: 10.1128/mbio.00920-24 (PMC11237595; doi:10.1128/mbio.00920-24)
Supplement: Supplemental material — Figures S1-S8 and Tables S1-S2. [file mbio.00920-24-s0001.pdf]

Aspartyl peptidase May1 induces host inflammatory response by altering cell wall composition in the fungal pathogen *Cryptococcus neoformans*

Running title: May1 remodels cryptococcal cell wall in acidic environment

Yeqi Li[1], Benjamin Chadwick[2], Tuyetnhu Pham[2], Xiaofeng Xie[1], Xiaorong Lin[1,2*]

[1]Department of Microbiology, University of Georgia, Athens, Georgia, USA

[2]Department of Plant Biology, University of Georgia, Athens, Georgia, USA

* Correspondence: xiaorong.lin@uga.edu

Keywords: carbon dioxide, pH sensing, chitosan, chitin synthase, chitin deacetylase, inflammation, cryptococcosis

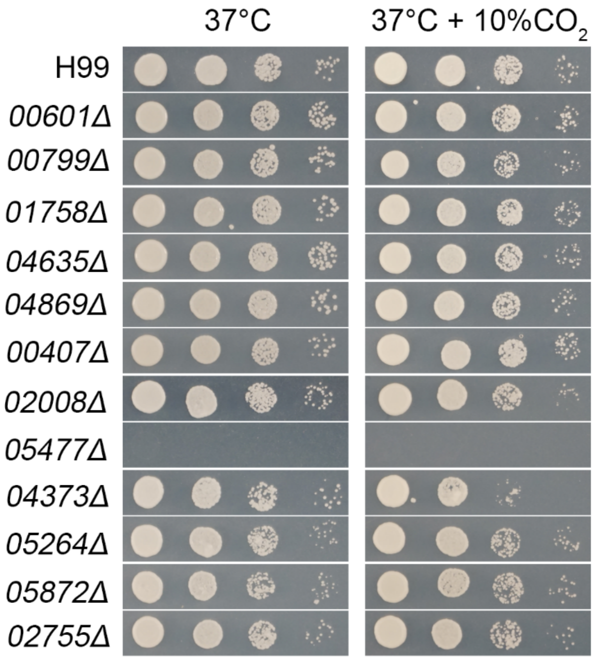

Fig. S1 CO_2 sensitivity of the knockout mutants of the putative candidate genes. The indicated mutant strains and the wild-type H99 control strain were spotted onto the YNB medium and incubated at 37ºC in ambient air or in 10% CO_2 for 2 days.

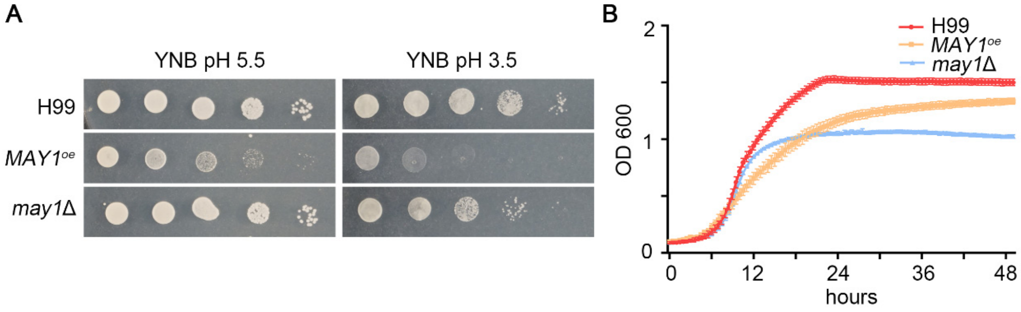

Fig. S2 Acidic pH causes the growth defect of the *MAY1* deletion and overexpression strains.

(A) The indicated strains were spotted onto the YNB (pH 5.5) or the YNB (pH 3.5) media and incubated at 37°C for 2 days. (B) The overnight cultures of H99, the *may1*Δ and the *MAY1^{oe}* strains were diluted to OD_{600} = 0.1 in YNB medium, inoculated into a 24-well microplate, and incubated at 37 °C with double orbital shaking in a Biotek Epoch 2 plate reader. Three replicates of each strain were included. Growth was monitored every 30 min by measuring OD_{600}.

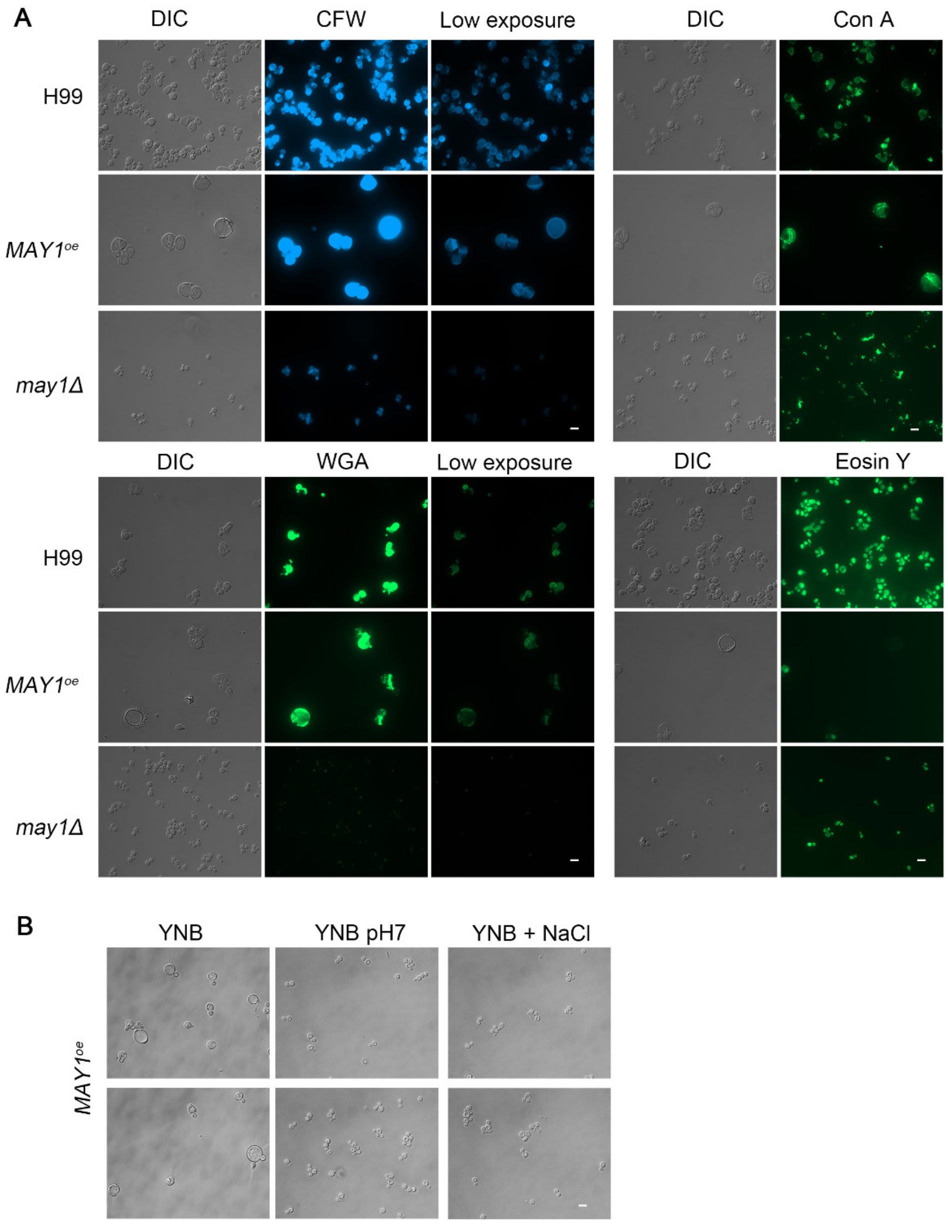

Fig. S3 May1 activities enlarge cell size and alter cell wall compositions.

(A) Cells of the wild-type H99, the *MAY1*^{oe} and the *may1*Δ strains were grown in liquid YNB medium for 48 h and then stained with CFW, ConA, WGA, or EosinY. Representative images are shown. Scale bar: 10 µm. (B) Cells of the *MAY1*^{oe} strain were cultured in liquid unbuffered YNB medium, YNB medium buffered to pH7, or unbuffered YNB supplemented with 0.25 M NaCl. Cells were incubated at 37ºC for 2 days. Scale bar: 20 µm.

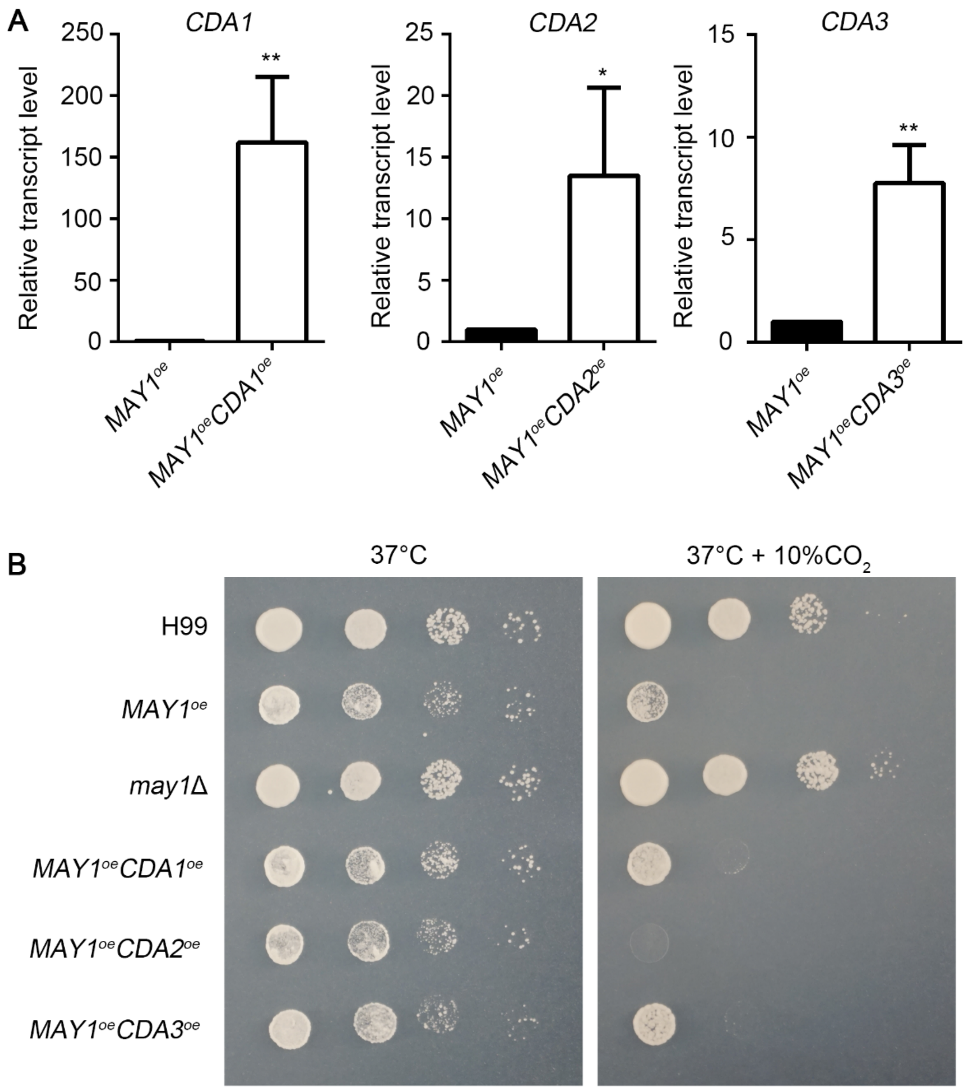

Fig. S4 Overexpression of *CDA1, CDA2,* or *CDA3* does not rescue the growth defect of the *MAY1*oe strain in high levels of CO_2.

(A) The relative transcript levels of *CDA1*, *CDA2* and *CDA3* in the indicated strains were measured by RT-PCR. The transcript level of the house-keeping gene *TEF1* was used as the internal control in every sample. The transcript level of each gene was compared to that in the parent strain, which was set to 1 for normalization. The experiments were performed in three independent biological replicates. (B) Cells of the indicated strains were serial diluted, spotted onto the YNB medium and incubated at 37ºC in ambient air or in 10% CO_2 for 2 days. Statistical significance was determined using a one-way ANOVA statistical analysis. *: $p < 0.05$; **: $p < 0.01$.

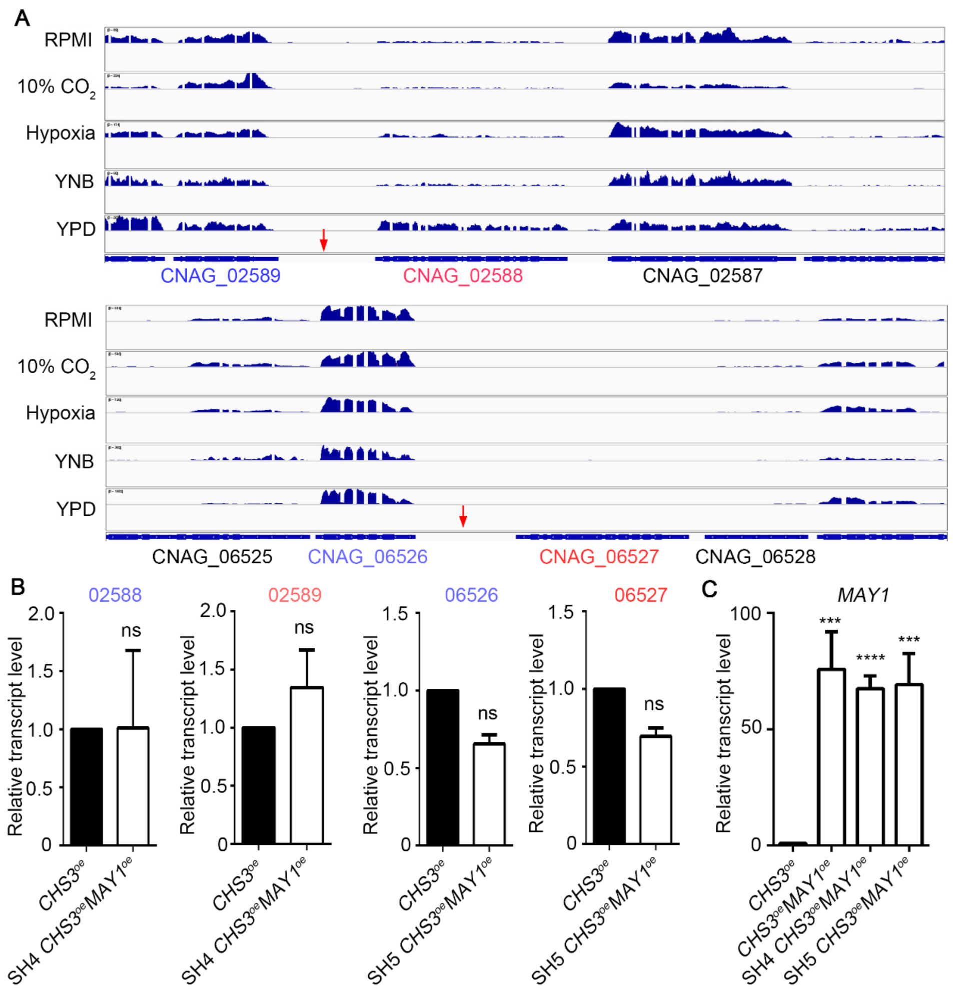

Fig. S5 Identification of two additional safe haven sites in *C. neoformans* H99. (A) A genome browser shot of the transcript profiles for the two potential safe haven candidates based on the published RNA-seq data. The red arrows indicate the designed guide RNA target site used in this study. (B) The relative transcript levels of genes neighboring the two candidate sites in the indicated strains were measured by RT-PCR. (C) The relative transcript levels of *MAY1* in the indicated strains were measured by RT-PCR. The transcript level of the house-keeping gene *TEF1* was used as the internal control in every sample. The transcript level of each gene was compared to that in the parent strain, which was set to 1 for normalization. The experiments were performed in three independent biological replicates. Statistical significance was determined using a one-way ANOVA statistical analysis. ns: not significant; ***: $p < 0.001$; ****: $p < 0.0001$.

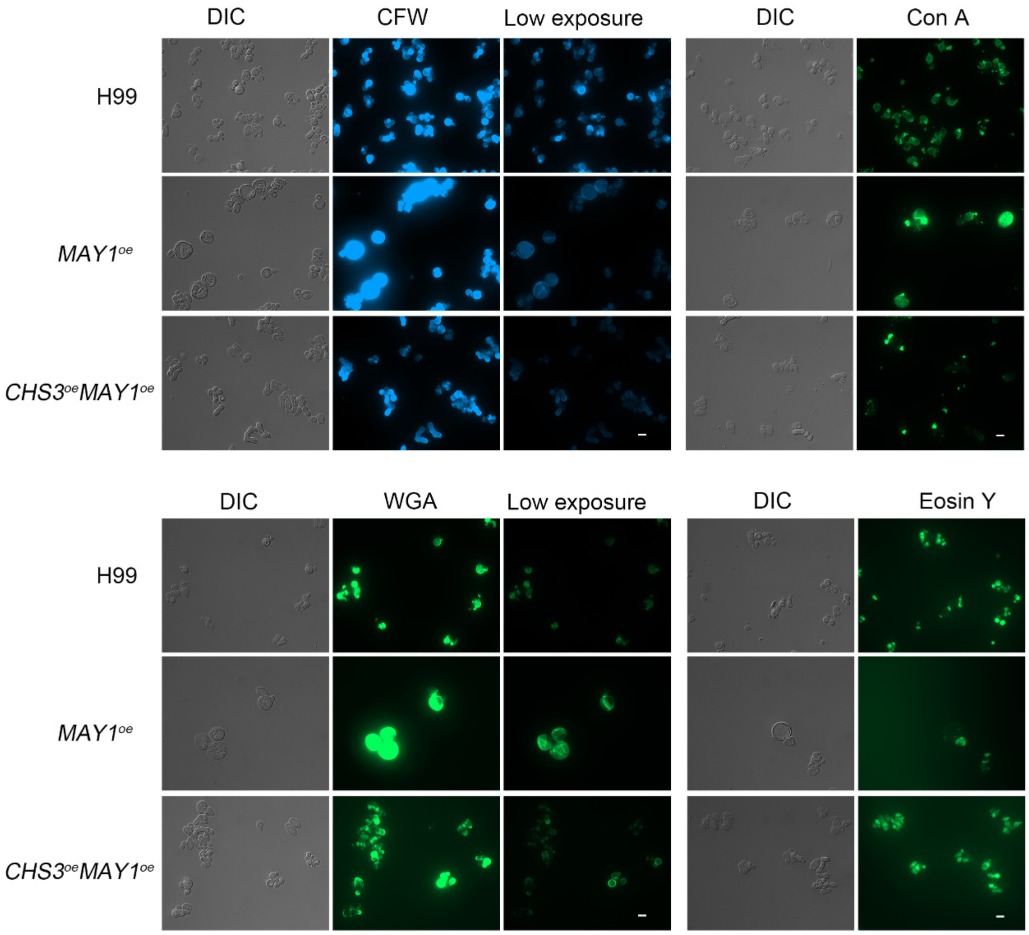

Fig. S6 Overexpression of *CHS3* rescues the chitosan deficiency of the *MAY1*^{oe} strain. Cells of the indicated strains were grown in liquid YNB medium for 48 hrs and then stained with CFW, ConA, WGA or EosinY. Representative images are shown. Scale bar: 10 μm.

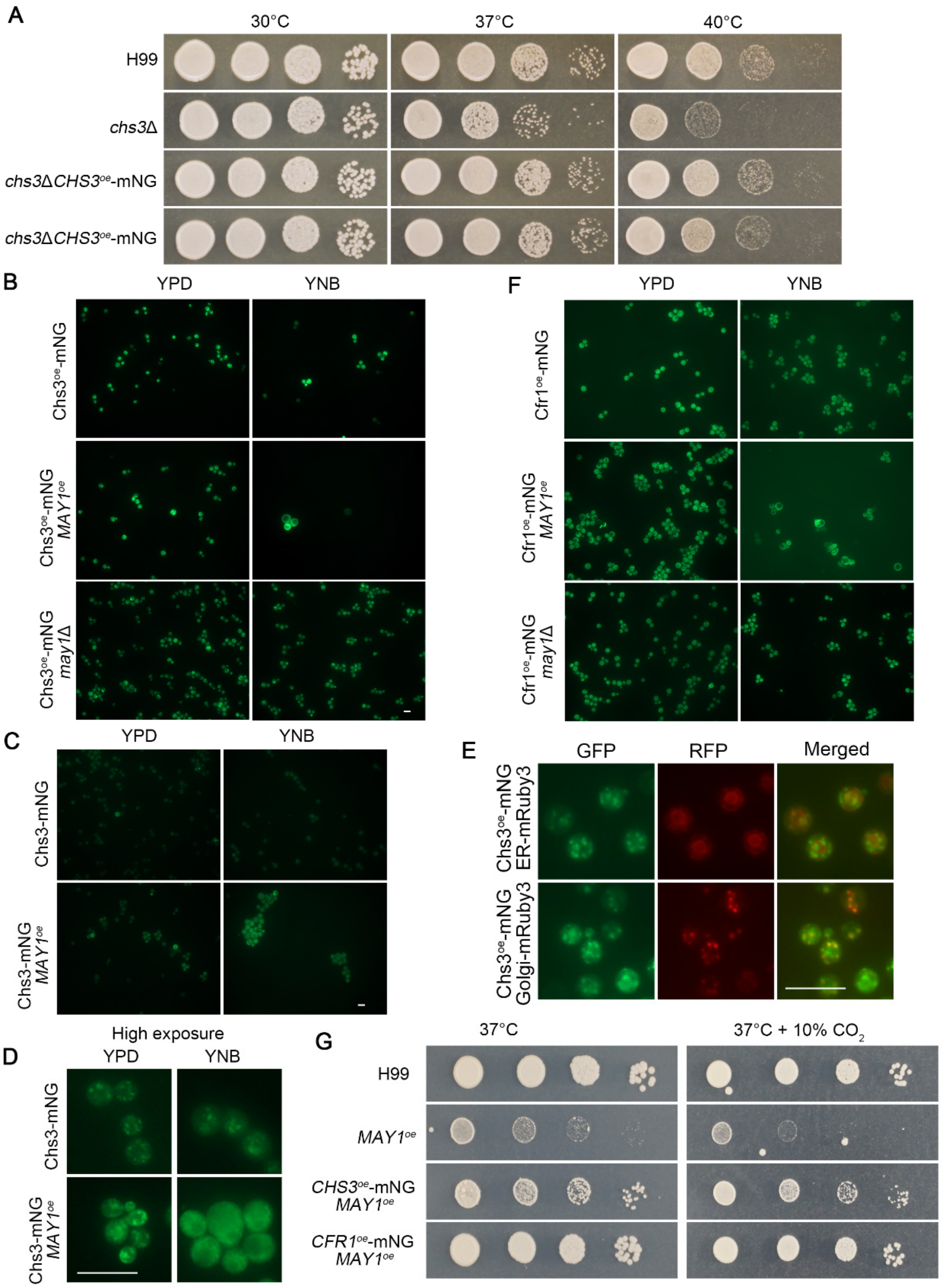

Fig. S7 Overexpression of *MAY1* reduces Chs3 puncta.

(A) The indicated strains were spotted onto the YPD medium and incubated at 30ºC, 37ºC, or 40ºC for 2 days. (B) The Chs3 fluorescently tagged strains (in H99 and the *MAY1*^{oe} strain background) were cultured in liquid YPD or YNB medium at 37ºC for 2 days. (C-D) The native promoter driven Chs3 fluorescently tagged strains were cultured in liquid YPD or YNB medium at 37ºC for 2 days. (D) The increased

exposure time of cells from panel (C). (E) The indicated strains with fluorescently tagged Chs3 and fluorescently marked ER or Golgi were cultured in liquid YPD at 37ºC for 1 day. (F) The Cfr1 fluorescently tagged strains were cultured in liquid YPD or YNB medium at 37ºC for 2 days. (G) Cells of the indicated strains were serial diluted, spotted onto the YNB medium, and incubated at 37ºC in ambient air or in 10% CO_2 for 2 days.

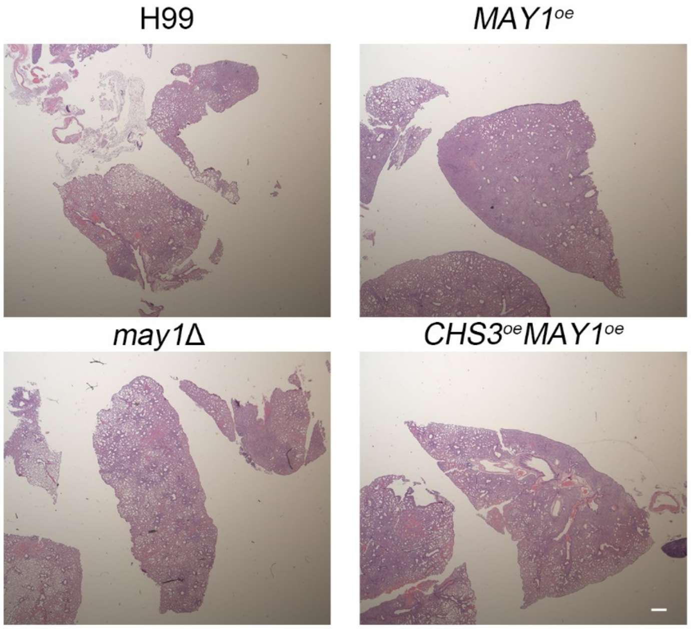

Fig. S8 May1 activities positively correlate with the host hyper-inflammatory response.

H&E staining of the lungs of mice inoculated with HK cells of the indicated strains at DPI 3.

Table S1. The selected candidates encoding predicted secretory proteins from the RNA-seq analysis

Gene ID	Product Description	SignalP Peptide	Predicted localization
CNAG_00407	Glyoxal oxidase	MYSAILLSLLPLLAAA	Extracellular
CNAG_00601	Glycosyl hydrolase	MLFPALALLCPVLVAA	Extracellular
CNAG_00799	Cellulase	MFAFTYIAALLSLISVLPSALA	Extracellular
CNAG_01758	Polygalacturonase	MRFICLLVCALSSAVVEG	Extracellular
CNAG_02008	PEBP-like protein	MLGYFAALALALPALAQS	Extracellular
CNAG_02775	BCS-inducible membrane protein	MFALKSILVTSLITSTALA	Extracellular
CNAG_03176	Endoplasmic oxidoreductin 1	MRLTAKLAVLSLVAAVSA	Mitochondria
CNAG_04065	UDP-N-acetylglucosamine transferase subunit ALG13	MSHPFTLLATVGSTLFPSLTS	Extracellular
CNAG_04373	Alginate lyase	MLTSIIAALPLLASLPLSSA	Extracellular
CNAG_04635	Endopeptidase	MIAPLLLFLTLPLAFA	Extracellular
CNAG_04869	Para-nitrobenzyl esterase	MRWLNIFPLLLPFVADA	Extracellular
CNAG_05264	Alpha-amylase AmyA	MPVLSNLISFLPFLAIAHA	Extracellular
CNAG_05477	PProtein disulfide-isomerase	MRLPRLLSATFSLSALLTKATA	Mitochondria
CNAG_05872	Endopeptidase	MHYLAVALPLLTLALA	Extracellular

Table S2 Strains and primers used in this study.

Fungal Strains	SOURCE	IDENTIFIER
H99		*C. neoformans* serotype A, wild type, MATalpha,
Linlab9114	FGSC deletion set 14C8	MATalpha, *CNAG_00601::NAT*
Linlab9115	FGSC deletion set 2B9	MATalpha, *CNAG_00799::NAT*
Linlab9116	FGSC deletion set 9B7	MATalpha, *CNAG_01758::NAT*
Linlab9117	FGSC deletion set 4G12	MATalpha, *CNAG_04635*::NAT
Linlab9118	FGSC deletion set 8B11	MATalpha, *CNAG_00407::NAT*
Linlab9120	FGSC deletion set 3C8	MATalpha, *CNAG_04373::NAT*
Linlab9121	FGSC deletion set 3B8	MATalpha, *CNAG_05264::NAT*
Linlab9121	FGSC deletion set 17G10	MATalpha, *CNAG_05477::NAT*
Linlab7367	FGSC deletion set 5D10	MATalpha, *CNAG_05872::NAT*
Linlab353	doi:10.1128/IAI.00296-12	A1-38-2, A1/M1, molecular type VNI
Linlab349	doi:10.1128/IAI.00296-12	A7-35-23, vnii**, molecular type VNII
Linlab6274	This manuscript	MATalpha, *CNAG_02008::NAT*
Linlab7361	This manuscript	MATalpha, *CNAG_02775::NAT*
Linlab7232	This manuscript	MATalpha, P_{TEF1}-*CNAG_05872-NEO*
Linlab8590	This manuscript	MATalpha, P_{TEF1}-*CNAG_05581-NAT*

Linlab8822	This manuscript	MATalpha, *P_{TEF1}-CNAG_05581-NAT, P_{TEF1}-CNAG_05872-NEO* (SH4)
Linlab8832	This manuscript	MATalpha, *P_{TEF1}-CNAG_05581-NAT, P_{TEF1}-CNAG_05872-NEO* (SH5)
Linlab8835	This manuscript	MATalpha, *P_{TEF1}-CNAG_05581-NAT, P_{TEF1}-CNAG_05872-NEO* (Ectopic)
Linlab	This manuscript	*MATa, P_{TEF1}-CNAG_05581-mNG-NEO*
Linlab9250	This manuscript	*MATa, P_{TEF1}-CNAG_05581-mNG-NEO, P_{TEF1}-CNAG_05872-HYG*
Linlab	This manuscript	*MATa, P_{TEF1}-CNAG_04321-mNG-NEO*
Linlab9313	This manuscript	*MATa, P_{TEF1}-CNAG_04321-mNG-NEO, P_{TEF1}-CNAG_05872-HYG*
Linlab8825	This manuscript	MATalpha, *P_{TEF1}-CNAG_05799-NEO, P_{TEF1}-CNAG_05799-NEO*
Linlab8928	This manuscript	MATalpha, *P_{TEF1}-CNAG_05799-NEO, P_{TEF1}-CNAG_01230-NEO*
Linlab8634	This manuscript	MATalpha, *P_{TEF1}-CNAG_05799-NEO, P_{TEF1}-CNAG_01239-NEO*
Linlab9685	This manuscript	*P_{TEF1}-CNAG_05581-NEO, P_{TEF1}-ER-mRuby3-HYG*
Linlab9687	This manuscript	*P_{TEF1}-CNAG_05581-NEO, P_{TEF1}-Golgi-mRuby3-HYG*
Linlab9691	This manuscript	MATalpha, *CNAG_05581::NAT, P_{GPD1}-CNAG_05581-mNG-NEO*
Linlab9698	This manuscript	MATa, *P_{CNAG_05581}-CNAG_05581-mNG-NEO*
Linlab9712	This manuscript	MATa, *P_{GPD1}-CNAG_05581-flag-HYG*
Linlab9715	This manuscript	MATalpha, *P_{TEF1}-CNAG_05872-NEO, P_{GPD1}-CNAG_05581-flag-HYG*
Linlab9802	This manuscript	MATalpha, *CNAG_05872::NAT, P_{GPD1}-CNAG_05581-mNG-NEO*
Linlab9810	This manuscript	MATalpha, *CNAG_05872::NAT, P_{GPD1}-CNAG_05818-mNG-NEO*
Linlab9828	This manuscript	MATa, *P_{CNAG_05581}-CNAG_05581-mNG-NEO, P_{GPD1}-CNAG_05872-HYG*
Primers		
M13F	GTAAAACGACGGCCAG	
M13R	CAGGAAACAGCTATGAC	
Linlab6033	CCACAGAAAACTTCAAAGGCCGGCCCCACAATGCACTACCTCG	MAY1-F-FseI
Linlab6034	CTACTGCTACTGTAACCCTTAATTAACTCTAGATCATTGCGGCAAC	MAY1-R-PacI
Linlab8589	AGAAAACTTCAAAGGCCGGCCATGTCGCGACCCCATCTCC	CHS3-F-FseI

Linlab8590	TGCTACTGTAACCCTTAATTAATTAATACTGGGGAGGCGC	CHS3-R-PacI
Linlab9316	atagtgggagacatatcttaAACAGTATACCCTGCCGGTG	SH4-sgRNA-U6promoter
Linlab9317	taagatatgtctcccactatGTTTTAGAGCTAGAAATAGCAAGTT	SH4-sgRNA-scaffold
Linlab9318	ccaaacgcgcaggctgttctAACAGTATACCCTGCCGGTG	SH5-sgRNA-U6promoter
Linlab9319	agaacagcctgcgcgtttggGTTTTAGAGCTAGAAATAGCAAGTT	SH5-sgRNA-scaffold
Linlab9096	gaagcgttcaagttttg	SH4 screening-F
Linlab9097	gctggtgatgaggcagg	SH4 screening-R
Linlab9098	CTACTTCAACTGCTACGT	SH5 screening-F
Linlab9099	AAAATAGTAATAGCAGC	SH5 screening-R
Linlab9316	atagtgggagacatatcttaAACAGTATACCCTGCCGGTG	SH4-sgRNA-U6promoter
Linlab9317	taagatatgtctcccactatGTTTTAGAGCTAGAAATAGCAAGTT	SH4-sgRNA-scaffold
Linlab9318	ccaaacgcgcaggctgttctAACAGTATACCCTGCCGGTG	SH5-sgRNA-U6promoter
Linlab9319	agaacagcctgcgcgtttggGTTTTAGAGCTAGAAATAGCAAGTT	SH5-sgRNA-scaffold
Linlab5714	TAGCCAggccggccCCATGTTTACATTCGCTGCC	CDA1-F-FseI
Linlab5715	GGttaattaaTTAGGCGACATATACCATACC	CDA1-R-PacI
Linlab5523	TAGCCAggccggccATGATCCCTTCCACCGCC	CDA2-F-FseI
Linlab5524	GGttaattaaTTACAGCAGCATCACACCAG	CDA2-R-PacI
Linlab5525	TAGCCAggccggccATGTACGGTCATTTATCTCTCTCC	CDA3-F-FseI
Linlab5526	GGttaattaaTCAGACCATTATAGCAGCAAGGG	CDA3-R-PacI
Linlab6782	AGAATGTCTACACCGCTTTC	CNAG_05872 RT PCR F
Linlab6783	CACACCCTTCCTCTCCATAG	CNAG_05872 RT PCR R
Linlab6780	AAGTCTCTGCACCTTCTGA	CNAG_05799 RT PCR F
Linlab6781	ACATATACCATACCAACCGC	CNAG_05799 RT PCR R
Linlab6724	ACTCAGAAAGATGGAAGCAG	CNAG_01230 RT PCR F
Linlab6725	ACTACACCCCCCAAAATAAG	CNAG_01230 RT PCR R

Linlab6726	TACCTATGTCGCTTCCTCAA	CNAG_01239 RT PCR F
Linlab6727	AGACCATTATAGCAGCAAGG	CNAG_01239 RT PCR R
Linlab8775	TGGTTCAGCGAGCCAACTCA	CNAG_05581 RT PCR F
Linlab8776	GGCCGTACCAAGTCCACTGT	CNAG_05581 RT PCR R
